# Health-Related Quality of Life of Adolescents and Children With Type 1 Diabetes in the Jazan Region of Saudi Arabia

**DOI:** 10.7759/cureus.53307

**Published:** 2024-01-31

**Authors:** Gassem A Gohal, Aqilah Majhali, Esaam Moafa, Sarah H Talebi, Bushra I Maashi, Amani Mutaen, Walaa J Alhamdan, Ibrahim M Dighriri

**Affiliations:** 1 Department of Pediatrics, Faculty of Medicine, Jazan University, Jazan, SAU; 2 Department of Medicine, Faculty of Medicine, Jazan University, Jazan, SAU; 3 Department of Internal Medicine, Faculty of Medicine, Jazan University, Jazan, SAU; 4 Department of Medicine, Medical University of Warsaw, Warsaw, POL; 5 Department of Pharmacy, King Abdulaziz Specialist Hospital, Taif, SAU

**Keywords:** pediatric diabetes, type 1 diabetes mellitus (t1d), health-related quality of life, worry, treatment barriers, diabetes symptoms, hrqol, t1dm

## Abstract

Background: Type 1 diabetes mellitus (T1DM) is increasingly prevalent among Saudi Arabian youth, particularly in the Jazan region. This chronic condition necessitates lifelong insulin therapy and poses significant daily management challenges for affected adolescents. Despite the high incidence rates, there is a notable lack of research into how T1DM impacts the health-related quality of life (HRQoL) of these individuals.

Objective: This study aimed to assess HRQoL and its demographic correlates in T1DM patients in the Jazan region of Saudi Arabia.

Methods: In this cross-sectional study, 236 T1DM patients completed the Pediatric Quality of Life Inventory Diabetes Module 3.0 (PedsQL DM). The HRQoL across domains of diabetes symptoms, treatment barriers, adherence, worry, and communication was compared by gender, nationality, age, education, residence, and healthcare follow-up using t-tests and ANOVA. Multivariate regression identified predictors of overall HRQoL.

Results: Most respondents were female (51.3%), 42.8% were between the ages of seven and 12 years, and 94.5% were Saudi nationals. Males reported better HRQoL than females, with fewer symptoms, treatment barriers, and better communication (all p<0.05). Non-Saudis had better treatment adherence, communication, and overall HRQoL than Saudis (all p<0.05). Older children (13-18 years) reported lower treatment barriers than younger children (three to six years) (p<0.05). Those with intermediate education had lower treatment barriers than those with preliminary education (p = 0.038). Only the female gender (-0.171, p = 0.009) independently predicted poorer overall HRQoL.

Conclusion: This study revealed disparities in HRQoL among T1DM children and adolescents. Males, non-Saudis, older children, and those with more education had better HRQoL. Females were at particular risk for poorer outcomes. Targeted interventions are needed to address this region's demographic disparities in diabetes-related HRQoL.

## Introduction

Diabetes is a global epidemic affecting 382 million people worldwide and is projected to rise to 582 million by 2035 [1]. This public health crisis particularly impacts Saudi Arabia, where 24% of the population lives with the disease [2, 3]. The prevalence of type 1 diabetes mellitus (T1DM) is notably high among children and adolescents, having more than doubled in the past decade to an average incidence of 27.2 per 100,000 people [2, 3]. Type 1 diabetes mellitus is marked by chronic hyperglycemia due to complete insulin deficiency, requiring lifelong insulin replacement therapy [4,5].

Adolescents with T1DM face many daily challenges, such as stringent insulin therapy regimens, dietary restrictions, regular exercise, and frequent biochemical marker monitoring [6-8]. Historically, T1DM management has concentrated on maintaining reasonable metabolic control to prevent long-term complications [9, 10]. Health-related quality of life (HRQoL) has emerged as a pivotal aspect of patient care [11, 12]. Health-related quality of life encapsulates the impact of an individual's health status on their life quality across physical, psychological, and social domains [11, 12]. The importance of HRQoL is particularly amplified in T1DM, where the disease can significantly impact adolescents undergoing complex physical, emotional, and social transitions [8, 12].

Adolescents with T1DM have been reported to possess the poorest HRQoL among all patient groups [13, 14]. They encounter numerous psychological challenges, including an increased risk of depression [15, 16]. The distinct needs of adolescents, underscored by the significant developmental changes during this transitional phase, add complexity to T1DM management [17, 18]. Moreover, family relationships and parental support are vital in managing T1DM [8, 18].

Despite the high prevalence of T1DM among children and adolescents in Saudi Arabia, especially in the Jazan region [2], there is a conspicuous gap in the literature assessing the HRQoL of adolescents with T1DM in this demographic. This study aims to fill that void by examining the HRQoL of adolescents under 18 years of age living with T1DM in the Jazan region. This study was planned to assess the impact of T1DM on various aspects of adolescents' lives and identify the factors influencing these HRQoL outcomes. Understanding the HRQoL of adolescents with T1DM is imperative to guide the creation of targeted interventions and support services to enhance their well-being. The anticipated findings from this study are expected to significantly contribute to the growing body of knowledge on T1DM adolescents’ HRQoL. Additionally, this research will shape future investigations and influence healthcare policies in the Jazan region and beyond.

## Materials and methods

Study design and study settings

This study was cross-sectional and was conducted from March to May 2023 in Jazan. Jazan region, also referred to as Jizan or Gizan is a southwestern province of Saudi Arabia. It is one of the country's 13 administrative divisions, located along the coast of the Red Sea. A significant portion of the population, around 60%, resides in rural settings. The most populous city and the regional capital is Jazan, which has approximately 300,000 residents.

Study population

Adolescents and children in the Jazan region below the age of 18 who had been diagnosed with T1DM for over a year formed the study population.

Inclusion criteria

The inclusion criteria were adolescents and children under the age of 18 residing in the Jazan region who have been diagnosed with T1DM for at least one year.

Exclusion criteria

Individuals over 18 years of age, non-residents of the Jazan region, those suffering from other chronic diseases, and non-consenting participants in the study were excluded.

Sampling method and sample size

The research utilized a simple random technique to guarantee equitable representation of the populace, minimizing sampling bias. The Raosoft calculator (Raosoft Inc., Seattle, WA) [19] was employed to ascertain the sample size. Based on a 95% confidence level, a margin of error of 5%, an anticipated response rate of 50%, and a total diabetic patient count of 39,000 in Jazan [20], the smallest required sample size was determined to be 381.

Data collection tools and processes

Information was gathered through an online, validated, self-administered survey facilitated by various data collectors. The questionnaire was based on the Pediatric Quality of Life Diabetes Module 3.0 (PedsQL DM) [21] and comprised the following sections: Diabetes symptoms (11 questions): This scale assesses common symptoms and complications associated with diabetes, such as frequent urination, extreme thirst, and fatigue; Treatment barriers (four questions): This scale addresses difficulties in managing diabetes treatment, such as remembering to take medication or checking blood glucose levels; Treatment adherence (seven questions): This scale assesses how well the child or adolescent follows their treatment plan, such as taking medication as prescribed and following dietary guidelines; Worry (three questions): This scale assesses concerns related to having diabetes, such as worrying about blood sugar levels or future complications; Communication (three questions): This scale assesses communication with healthcare providers, such as feeling comfortable asking questions or discussing concerns.

Each item is scored on a five-point scale from 0 (never a problem) to five (almost always a problem). The scores are then reversed and transformed into a scale, with higher scores indicating better HRQoL. Total HRQoL was calculated as the sum scores of diabetes symptoms plus treatment barriers plus treatment adherence plus worry plus communication

Ethical considerations

Jazan University's Scientific Research Ethics Committee (REC) in Jazan, Saudi Arabia, under approval number REC-44/07/544, granted ethical clearance on February 5, 2023. Participants were also given a digitally accessible information letter for their electronic consent. This was displayed as an initial page before starting the online questionnaire. The research process placed significant emphasis on respecting the dignity of all participants.

Statistical analysis

The study utilized the IBM Statistical Package for the Social Sciences (SPSS) software for data analysis (IBM Corp., Armonk, NY). Initially, data were gathered from participants via Google Forms (Google Inc., Mountainview, CA), documenting characteristics such as age, gender, nationality, education level, place of residence, and health center follow-up status. After data collection, the responses were transferred to Microsoft Excel (Microsoft Corp., Redmond, WA) for cleaning and coding and subsequently imported into SPSS for comprehensive statistical analysis. Descriptive analysis was performed to generate participant demographics, followed by independent t-tests to determine significant differences in diabetes-related experiences and HRQoL based on gender, nationality, living place, and health center follow-ups. A one-way ANOVA was employed to examine differences based on age groups and education levels. In cases of significant results, Tukey post hoc tests were conducted to pinpoint the specific groups with significant differences. The study also utilized multiple linear regression analysis to identify the factors predicting HRQoL, considering the total HRQoL score as the dependent variable and age, gender, nationality, education level, living place, and health center follow-up status as independent variables. A p-value <0.05 was considered indicative of statistical significance for all the tests carried out. The results, including mean scores and standard deviations for various PedsQL DM scales, were then meticulously reported in the study's results section.

## Results

This study included 236 participants, of whom the majority were female (51.3%). Regarding age, 14.8% of participants were between three and six years old, 42.8% were between the ages of seven and 12, and 42.4% were between the ages of 13 and 18. Most participants were Saudi nationals (94.5%), with 5.5% being non-Saudi. Regarding education level, 12.7% had a preliminary education, 36.0% had a primary education, 29.2% had an intermediate education, 17.8% had a secondary education, 3.4% had a collegiate education, and 0.8% were illiterate. The sample was predominantly from villages (76.7%), with 23.3% living in cities. Finally, most participants (92.4%) reported being followed up at a health center, while 7.6% did not (Table 1).

**Table 1:** Sociodemographic characteristics of the participants N: number

Variable	Category	N	Percentage (%)
Gender	Male	115	48.7
	Female	121	51.3
Age (in years)	3–6	35	14.8
	7–12	101	42.8
	13–18	100	42.4
Nationality	Saudi	223	94.5
	Non-Saudi	13	5.5
Education	Preliminary	30	12.7
	Primary	85	36.0
	Intermediate	69	29.2
	Secondary	42	17.8
	College	8	3.4
	Illiterate	2	0.8
Place of residence	City	55	23.3
	Village	181	76.7
Do you follow up at a health center?	Yes	218	92.4
	No	18	7.6

An independent t-test for the PedsQL DM with gender is shown in Table 2.

**Table 2:** Analysis of the PedsQL DM with gender N: number; PedsQL DM: Pediatric Quality of Life Inventory Diabetes Module; SD: standard deviation; HRQoL: health-related quality of life; p < 0.05 is considered significant

PedsQL DM	Gender	N	Mean	SD	p-value
Diabetes symptoms	Male	115	29.7652	6.73213	0.038
	Female	121	27.8926	7.04722	
Treatment barrier	Male	115	11.2783	3.67435	0.009
	Female	121	10.0331	3.62384	
Treatment adherence	Male	115	21.5130	4.82749	0.059
	Female	121	20.1983	5.75126	
Worry	Male	115	7.0261	3.22126	0.562
	Female	121	6.7934	2.93518	
Communication	Male	115	9.8174	2.82092	0.031
	Female	121	8.9421	3.33990	
Total HRQoL	Male	115	79.4000	14.65378	0.007
	Female	121	73.8595	16.43285	

Analysis revealed significant gender differences in several areas of the PedsQL DM. For the diabetes symptoms scale, males reported a higher mean score (29.77 ± 6.73) than females (27.89 ± 7.05), indicating that males perceive fewer diabetes-related symptoms. This difference was statistically significant (p = 0.038). Regarding the treatment barrier scale, male participants also reported a higher mean score (11.28 ± 3.67) than their female counterparts (10.03 ± 3.62). This result suggests that males experience fewer barriers to treatment, and this difference was statistically significant (p = 0.009). For the treatment adherence scale, although males had a slightly higher mean score (21.51 ± 4.83) than females (20.20 ± 5.75), the difference was not statistically significant (p = 0.059). On the worry scale, the mean score for males was 7.03 ± 3.22, and for females, it was 6.79 ± 2.94. This indicates similar levels of worry between male and female participants about their diabetes, with no significant difference (p = 0.562). In the communication scale, males had a significantly higher mean score (9.82 ± 2.82) than females (8.94 ± 3.34), suggesting that males are better able to communicate about their diabetes (p = 0.031). Lastly, when it comes to the overall HRQoL, male participants reported a significantly higher mean score (79.40 ± 14.65) than females (73.86 ± 16.43), indicating that males have a better perceived HRQoL (p = 0.007).

The differences in diabetes-specific quality of life based on nationality using the PedsQL DM are shown in Table 3.

**Table 3:** Analysis of the PedsQL DM with nationality N: number; PedsQL DM: Pediatric Quality of Life Inventory Diabetes Module; HRQoL: health-related quality of life; SD: standard deviation; p < 0.05 is considered significant

PedsQL DM	Nationality	N	Mean	SD	p-value
Diabetes symptoms	Saudi	223	28.6323	6.80482	0.114
	Non-Saudi	13	31.7692	8.81432	
Treatment barrier	Saudi	223	10.6188	3.69965	0.718
	Non-Saudi	13	11.0000	3.71932	
Treatment adherence	Saudi	223	20.6278	5.35249	0.012
	Non-Saudi	13	24.4615	3.95001	
Worry	Saudi	223	6.9327	3.03730	0.592
	Non-Saudi	13	6.4615	3.75534	
Communication	Saudi	223	9.2601	3.15294	0.027
	Non-Saudi	13	11.2308	1.73944	
Total HRQoL	Saudi	223	76.0717	15.59234	0.049
	Non-Saudi	13	84.9231	17.69869	

Independent sample t-tests revealed no significant difference between Saudi and non-Saudi participants on the diabetes symptoms scale (p = 0.114), the treatment barrier scale (p = 0.718), or the worry scale (p = 0.592). However, statistically significant differences were found on the treatment adherence scale (p = 0.012), communication scale (p = 0.027), and total HRQoL score (p = 0.049). Specifically, non-Saudi participants reported better treatment adherence (M = 24.46, SD = 3.95) than Saudi participants (M = 20.63, SD = 5.35). Non-Saudi participants also communicated better (M = 11.23, SD = 1.74) than Saudi participants (M = 9.26, SD = 3.15). Finally, non-Saudis had higher total HRQoL scores (M = 84.92, SD = 17.70) than Saudis (M = 76.07, SD = 15.59).

Table 4 shows differences in diabetes-specific quality of life based on the place of residence (city vs. village) using the PedsQL DM.

**Table 4:** Analysis of the PedsQL DM with the place of residence N: number; PedsQL DM: Pediatric Quality of Life Inventory Diabetes Module; SD: standard deviation; HRQoL: health-related quality of life; p < 0.05 is considered significant

PedsQL DM	Living	N	Mean	SD	p-value
Diabetes symptoms	City	55	29.2909	7.44280	0.555
	Village	181	28.6575	6.80064	
Treatment barrier	City	55	11.3091	3.53225	0.125
	Village	181	10.4365	3.72717	
Treatment adherence	City	55	20.6727	5.81204	0.793
	Village	181	20.8895	5.21844	
Worry	City	55	7.1273	3.16845	0.545
	Village	181	6.8398	3.04991	
Communication	City	55	9.6000	3.10078	0.531
	Village	181	9.2983	3.13394	
Total HRQoL	City	55	78.0000	16.56972	0.441
	Village	181	76.1215	15.58477	

Independent sample t-tests revealed no significant differences between participants from city and village on any of the PedsQL, which were diabetes symptoms (p = 0.555), treatment barriers (p = 0.125), treatment adherence (p = 0.793), worry (p = 0.545), communication (p = 0.531), or total HRQoL (p = 0.441). Participants living in cities had similar mean scores for all diabetes modules as those living in villages.

Independent sample t-tests revealed no significant differences between those who did versus did not follow up at a health center on any of the PedsQL DM scales: diabetes symptoms (p = 0.610), treatment barriers (p = 0.529), treatment adherence (p = 0.312), worry (p = 0.615), communication (p = 0.322), or total HRQoL (p = 0.314). Total HRQoL was comparable between those who did (M = 76.86, SD = 15.43) and did not (M = 72.94, SD = 19.96) follow up at a health center (Table 5).

**Table 5:** Analysis of the PedsQL DM with the participants' follow-up status at the health center PedsQL DM: Pediatric Quality of Life Inventory Diabetes Module; HRQoL: health-related quality of life; N: number; SD: standard deviation; p < 0.05 is considered significant

PedsQL DM	Follow up with the health center	N	Mean	SD	SD. error mean	p-value
Diabetes symptoms	Yes	218	28.8716	6.89032	0.46667	0.610
	No	18	28.0000	7.73837	1.82395	
Treatment barrier	Yes	218	10.6835	3.60693	0.24429	0.529
	No	18	10.1111	4.71405	1.11111	
Treatment adherence	Yes	218	20.9404	5.28376	0.35786	0.312
	No	18	19.6111	6.13705	1.44652	
Worry	Yes	218	6.9358	3.08639	0.20904	0.615
	No	18	6.5556	2.97484	0.70118	
Communication	Yes	218	9.4266	3.10739	0.21046	0.322
	No	18	8.6667	3.30774	0.77964	
Total HRQoL	Yes	218	76.8578	15.43032	1.04507	0.314
	No	18	72.9444	19.96017	4.70466	

A one-way ANOVA was conducted to examine differences in diabetes-specific quality of life based on age group (three to six years, seven to 12 years, and 13-18 years) using the PedsQL DM. There were no significant differences between the age groups on the diabetes symptoms, treatment adherence, worry, communication, and total HRQoL scales. However, a significant difference emerged for the treatment barrier scale. Post-hoc analyses revealed that 13-18-year-olds (M = 11.33) reported significantly lower treatment barriers and better quality of life than three- to six-year-olds (M = 8.40, p = 0.000). Additionally, three- to six-year-olds reported significantly higher treatment barriers and worse quality of life than seven- to 12-year-olds (M = 10.73, p = 0.003) (Table 6).

**Table 6:** Analysis of the PedsQL DM with age groups PedsQL DM: Pediatric Quality of Life Inventory Diabetes Module; HRQoL: health-related quality of life; p < 0.05 is considered significant

PedsQL DM	Age groups (years)	Mean difference	Significance	Mean (13-18 years)	Mean (3-6 years)	Mean (7-12 years)
Diabetes symptoms	13-18 vs 3-6	-0.07571	0.998	28.6100	28.6857	29.0396
	13-18 vs 7-12	-0.42960	0.900	28.6100	-	29.0396
	3-6 vs 7-12	-0.35389	0.964	-	28.6857	29.0396
Treatment barrier	13-18 vs 3-6	2.93000	0.000	11.3300	8.4000	10.7327
	13-18 vs 7-12	0.59733	0.464	11.3300	-	10.7327
	3-6 vs 7-12	-2.33267	0.003	-	8.4000	10.7327
Treatment adherence	13-18 vs 3-6	0.75429	0.754	21.2400	20.4857	20.5644
	13-18 vs 7-12	0.67564	0.645	21.2400	-	20.5644
	3-6 vs 7-12	-0.07864	0.997	-	20.4857	20.5644
Worry	13-18 vs 3-6	0.94571	0.261	7.0600	6.1143	7.0297
	13-18 vs 7-12	0.03030	0.997	7.0600	-	7.0297
	3-6 vs 7-12	-0.91542	0.283	-	6.1143	7.0297
Communication	13-18 vs 3-6	-0.08429	0.990	9.2300	9.3143	9.5248
	13-18 vs 7-12	-0.29475	0.783	9.2300	-	9.5248
	3-6 vs 7-12	-0.21047	0.937	-	9.3143	9.5248
Total HRQoL	13-18 vs 3-6	4.47000	0.322	77.4700	73.0000	76.8911
	13-18 vs 7-12	0.57891	0.963	77.4700	-	76.8911
	3-6 vs 7-12	-3.89109	0.422	-	73.0000	76.8911

A one-way ANOVA was conducted to examine differences in diabetes-specific quality of life based on education level using the PedsQL DM. There was no significant effect of education level on diabetes symptoms (p = 0.230), treatment adherence (p = 0.905), worry (p = 0.274), communication (p = 0.729), or total HRQoL (p = 0.767). However, education level significantly affected treatment barriers (p = 0.030). Tukey's post hoc test revealed that there was a significant difference between the intermediate education group and the preliminary education group for the dependent variable treatment barrier (p = 0.038). The intermediate education group had significantly lower treatment barriers than the preliminary education group (Table 7).

**Table 7:** Analysis of the PedsQL DM with education groups PedsQL DM: Pediatric Quality of Life Inventory Diabetes Module; DF: degrees of freedom; F: F-statistic; HRQoL: health-related quality of life; p < 0.05 is considered significant

PedsQL DM	Sum of squares	DF	Mean square	F	p-value
Diabetes symptoms	11333.034	235	48.597	1.388	0.230
Treatment barrier	3206.386	235	13.214	2.531	0.030
Treatment adherence	6727.881	235	29.054	0.313	0.905
Worry	2219.949	235	9.391	1.279	0.274
Communication	2290.928	235	9.840	0.562	0.729
Total HRQoL	58694.169	235	252.381	0.512	0.767

A multiple linear regression analysis was conducted to determine factors predicting HRQoL in the sample. The dependent variable was the total HRQoL score. The independent variables included in the analysis were age, gender, nationality, education level, living place, and whether participants were followed up at a health center. Of the predictor variables, only gender had a significant association with HRQoL. Being female was associated with a 5.403 lower HRQoL score than being male (β = -0.171, p = 0.009). Age, nationality, education level, living situation, and following up at a health center did not significantly predict HRQoL (p > 0.05) (Table 8).

**Table 8:** The dependent variable in regression analysis for all models is the total HRQoL SD. error (B): standard error of the unstandardized regression coefficient B; T: t-statistic; CI: confidence interval; HRQoL: health-related quality of life; p < 0.05 is considered significant.

Variable	Unstandardized coefficients (B)	SD. error (B)	Standardized coefficients (β)	t	p-value	95% CI (Lower)	95% CI (Upper)
Age	-0.964	1.753	-0.056	-0.550	0.583	-4.417	2.490
Gender	-5.403	2.056	-0.171	-2.628	0.009	-9.454	-1.352
Nationality	8.044	4.721	0.116	1.704	0.090	-1.258	17.347
Education	-0.976	1.264	-0.079	-0.772	0.441	-3.467	1.516
Living	-0.508	2.597	-0.014	-0.196	0.845	-5.625	4.608
Health center follow-up	-3.250	3.945	-0.055	-0.824	0.411	-11.023	4.523

## Discussion

This study delves into HRQoL assessment among adolescents and children diagnosed with T1DM in the Jazan region of Saudi Arabia, unearthing several significant findings. One salient observation was the existence of distinct HRQoL disparities based on gender, nationality, and age. An intriguing pattern emerged wherein male participants reported superior HRQoL compared to their female counterparts, echoing the outcomes of earlier studies [22-26]. This divergence could be attributed to the heightened perception of symptoms, communication challenges, and treatment hurdles that females encounter. Girls face additional stressors such as concerns over body image, self-esteem, and societal acceptance. Moreover, a fear of hypoglycemia can induce anxiety and restrict their activities, detrimentally impacting their quality of life [27]. Hormonal fluctuations that affect blood glucose levels exacerbate this fear, particularly for girls, impeding their participation in sports and other social events [28]. These factors collectively contribute to increased stress and diminished quality of life in females [27,29,30].

The data also revealed significant differences in HRQoL between Saudi and non-Saudi participants, with the latter group reporting improved treatment adherence, communication, and overall HRQoL. This intriguing finding necessitates further exploration to discern whether cultural discrepancies or socioeconomic factors underpin these disparities [30]. Age-wise, the research indicated that older adolescents aged 13-18 years (M = 11.33) reported significantly lower treatment barriers than younger age groups, indicating a better quality of life. The youngest group of children (three to six years, M = 8.40) experienced the highest treatment barriers, significantly more than the seven-to-12-year-olds (M = 10.73). These results align with studies suggesting that older adolescents may become more adept at managing their condition and more resilient in the face of treatment-related challenges [31]. On the other hand, the youngest children (three to six years) who reported the highest treatment barriers might be more reliant on their caregivers for diabetes management and therefore perceive greater barriers. This is supported by research indicating that younger children may feel more overwhelmed by the daily demands of diabetes care [32]. However, it is essential to note that no significant differences were found between the age groups regarding diabetes symptoms, treatment adherence, worry, communication, and total HRQoL scales.

Regarding education, the study highlighted that those in the intermediate education group faced fewer treatment barriers compared to the preliminary education group. This suggests that fostering a more comprehensive understanding of diabetes care in the intermediate stage could help reduce treatment barriers [33-35]. Contrary to some earlier studies [35, 36], our research found no discernible differences in HRQoL based on the place of residence (city vs. village) and regular health center follow-ups, despite the traditionally perceived lower HRQoL in rural areas due to limited healthcare access [35-37].

The analysis of multiple linear regression has pinpointed gender as the primary significant predictor of HRQoL, revealing that girls tend to report lower HRQoL scores compared to boys. This observation is in harmony with the outcomes of the t-test, underscoring the pivotal influence of gender on the management and treatment approaches for adolescent diabetes. The insights derived from this study are particularly valuable for understanding the HRQoL among children and adolescents with T1DM in the Jazan region. These insights lay the groundwork for designing interventions that are tailored to improve HRQoL within this specific population. There is a clear need for increased attention to bolster the quality of life for the groups identified as high-risk in this research. By doing so, healthcare providers and policymakers can work towards ensuring that these vulnerable groups receive the support they need to lead better-quality lives while managing their conditions.

Study limitations

In assessing the HRQoL of children and adolescents with T1DM in Saudi Arabia's Jazan region, this study revealed discernible disparities across various demographic groups. Contrasting male and female experiences, males reported higher HRQoL, perceived fewer diabetes symptoms, encountered fewer treatment barriers, and showed superior communication ability. When nationality was considered, non-Saudi participants presented better overall HRQoL outcomes compared to their Saudi counterparts. The study also observed a notable age-related disparity, where older children (13-18 years) faced fewer treatment barriers and demonstrated higher HRQoL than their younger counterparts (three to six years). Education emerged as another significant factor, with those with an intermediate education level encountering fewer treatment barriers than those with only a preliminary education. Interestingly, the living situation and frequency of healthcare follow-ups did not significantly impact HRQoL. However, gender was the predominant predictor of HRQoL, with female participants scoring significantly lower. The evidence of disparities in HRQoL outcomes among these groups signifies the need for targeted interventions, particularly for high-risk demographics such as females, younger children, Saudis, and those who are less educated. Future research should also explore other factors influencing the HRQoL of T1DM patients. Altogether, this study offers critical baseline data for understanding the disparities in HRQoL among children and adolescents with T1DM in the Jazan region of Saudi Arabia.

## Conclusions

This study found that among children and adolescents with T1DM in the Jazan region of Saudi Arabia, males reported a higher HRQoL than females, with fewer symptoms, treatment barriers, and better communication. Non-Saudis also had better HRQoL outcomes compared to Saudis. Interestingly, older children (13-18 years) faced fewer treatment barriers and had a higher HRQoL than younger children (three to six years). Those with intermediate education also had fewer treatment barriers than those with preliminary education. Gender was the main predictor of overall HRQoL, with females having significantly lower scores. No differences were seen based on the living situation or healthcare follow-up. These results indicate disparities in diabetes-related HRQoL, with males, non-Saudis, older children, and those with more education faring better. The findings highlight the need for targeted interventions to improve HRQoL among high-risk demographic groups like females, younger children, Saudis, and those with less education. Further research should explore additional factors influencing T1DM patients' lives. Overall, this study provides important baseline data on disparities in HRQoL in T1DM among Saudi children and adolescents in the Jazan region.
